# Performance of a Micro-Scale Membrane Reactor for Greywater Treatment at Household Level

**DOI:** 10.3390/membranes11010063

**Published:** 2021-01-17

**Authors:** Vasileios Diamantis

**Affiliations:** 1 Department of Environmental Engineering, Democritus University of Thrace, 67132 Xanthi, Greece; bdiamant@env.duth.gr; 2 Act4energy P.C., 45333 Ioannina, Greece

**Keywords:** micro-scale, wastewater treatment, greywater, microfiltration, membrane bioreactor, MBR, activated carbon, household

## Abstract

An aerated membrane reactor (25 L working volume) equipped with 1.5 m^2^ hollow-fiber module was designed and operated using synthetic greywater for household water reuse. Activated sludge (MBR), activated carbon (PAC), zeolite (ZEO) and iron hydroxide (GEH) were added in separate experiments to optimize membrane hydraulic performance and removal efficiency of organics. The use of additives improved permeate quality (in terms of Chemical Oxygen Demand—COD) compared to the direct membrane filtration mode of operation. GEH and MBR were efficient for phosphorus removal, which was not the case for PAC and ZEO. No significant improvement of membrane flux was recorded when PAC, ZEO or GEH were added inside the membrane tank. The MBR system displayed optimum performance during medium-term operation, with COD removal efficiency 85% and permeate flux between 40 and 25 L m^−2^ h^−1^. The capital costs of the proposed technology were around 300 € and the operational costs below 80 € yr^−1^, rendering the process feasible at household level. Greywater treatment systems for household applications are still on their infancy; however, this trend is expected to change due public perception towards circular economy, water conservation and reuse.

## 1. Introduction

Large-scale utilization of greywater as a renewable source of water will be clearly boosted by the penetration of micro-scale treatment systems at household level. Apart from manual collection of lightly polluted greywater (e.g., by using a bucket or bowl inside the wash basin) for reuse in toilet flushing or gardening, commercial treatment systems for household applications are still in their infancy. This trend; however, is expected to change due to the promotion of the circular economic model worldwide. Rational water management is among the most promising options to strategically plan climate change mitigation and adaptation [1]. On this basis, it is crucial to put forward technologies to exploit the greywater generated at household level.

Greywater reuse offers both economic and environmental advantages [2]. Indeed, it is a way to preserve freshwater resources and face-up water scarcity [3]. By reusing greywater it is possible to decrease household freshwater consumption by 10% to 40% [4]. This is important for regions with high water cost, such as small islands where the price per m^3^ can rise to 5–10 € [5]. Households without access to the sewer network (septic tanks) can also benefit by decreasing the quantities of wastewater generated. At municipality level, broad greywater reuse can lower the energy consumed for water and wastewater pumping [2,6]. Moreover, sewage will be less diluted, enabling the implementation of waste-to-energy technologies at centralized treatment plants [7,8].

For household greywater reuse, the development of micro-scale treatment technologies is of major importance. The design of such facilities should comply with limited space availability, high quality effluent and long term operational stability [9]. Another issue, highlighted by Vuppaladadiyam et al. [4] is the importance of continuous system maintenance, since unpleasant odors can be released inside the household. Cartridge filtration systems (such as activated carbon, zeolite, etc.) and membrane bioreactors were recently proposed by Jabornig and Favero [10] and Kant et al. [11]. Zeolites and activated carbon are well known to decrease ammonia and COD since they are characterized by cation exchange and adsorption capacity [12,13]. Widiastuti et al. [13] operated a zeolite percolation column and reported 75% COD removal efficiency comparable to activated carbon.

Membrane filtration systems are characterized by a small footprint (compact) and high degree of suspended and colloidal particles separation (including bacteria and viruses) [14]. Direct membrane filtration of greywater resulted in COD removal efficiency between 30% and 70% in accordance with the membrane pore size [11]. Besides, the dominant particle size in greywater samples was around 0.1 μm. Low COD removal efficiencies (between 20% and 30%) was reported during microfiltration of shower, wash basin and laundry effluents and this was attributed to the passage of soluble compounds, personal care products and household chemicals in the permeate [14]. The use of membranes for greywater treatment is very popular in the MBR configuration [9]. The latter is suitable for small-scale applications such as single-households, small housing complexes and residential buildings [2]. 

Membrane fouling during greywater treatment is of major concern. According to Nghiem et al. [15], the rapid flux decline in direct membrane filtration was attributed to the organics and calcium present in greywater. Other fouling components include particulate matter (organic and inorganic), surfactants and pathogens [2,4,14,16,17]. In order to minimize membrane fouling, it is; thus, necessary to decrease the concentration of organic foulants (particulate and soluble) inside the membrane tank [14]. This is possible by implementing either physical (e.g., adsorption) or biological processes. Adsorbents offer high stability toward harsh household chemicals (disinfectants, surfactants, bleach, etc.); however, they are characterized by a limited adsorption capacity which deteriorates reactor performance at the long-term. Moreover, the withdrawal and/or regeneration of the saturated material is an important barrier (practical and economical) for household applications.

Membrane bioreactors ensure the retention and biodegradation of both soluble and particulate organics, thus membrane fouling can be significantly decreased [2]. They are also effective in the removal of emerging contaminants and pharmaceutically active compounds from greywater [2]. According to Cecconet et al. [2] the MBR systems achieved surfactant removal efficiency higher than 80%, and in most of the cases higher than 97%. Clearly, it is possible to control membrane pore blocking and maintain high permeate flux by using adsorbents inside the MBR [18]. More information about membrane fouling in MBR systems can be found in recent review articles [18,19,20].

The aim of this study is to develop a micro-scale household greywater treatment system, based on the membrane reactor technology, for possible installation under the wash basin or kitchen sink. In detail the study aims to quantify: (a) Water usage and greywater generation for different household activities; (b) the effect of using additives (activated carbon, zeolite, iron hydroxide and activated sludge) on membrane hydraulic performance and permeate quality; and (c) the capital and operational costs of the proposed facility compared to similar systems reported in the literature.

## 2. Materials and Methods

### 2.1. Synthetic Greywater

Light greywater was prepared by diluting yogurt milk (ayran) (1 mL), fruit juice (1 mL) and cream soap (0.3 mL) per L tap water. By this addition the greywater was characterized as follow: pH = 6.44 (±0.10), electrical conductivity = 445 (±41) μS cm^−1^, carbohydrates = 130 mg L^−1^, proteins = 30 mg L^−1^, lipids = 15 mg L^−1^, COD = 230 (±35) mg L^−1^ and PO_4_-P = 0.54 (±0.28) mg L^−1^, similar to previous studies (e.g., [3,9,10,16]).

### 2.2. Frequency and Duration of Water Use at Student Residences

The frequency and duration of water use inside the household was recorded on daily basis by engineering students. The participants (n = 12) recorded the number of water uses per day and the duration of water use for different household activities. Additionally, they recorded the quantity (mL) of surfactant products used inside the wash basin and the kitchen sink. These values were considered for the design and operation of the proposed greywater treatment facility.

### 2.3. Membrane Reactor Design

**Figure 1:**
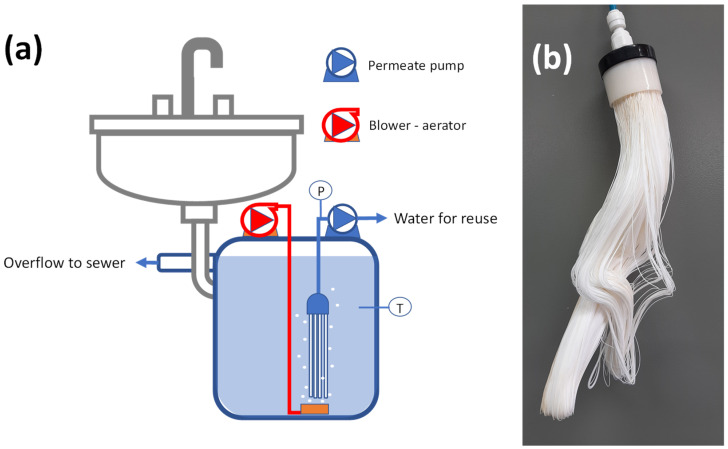
(**a**) Schematic representation of the experimental setup and (**b**) photographic representation of the hollow-fiber membrane module, used for this study.

The experimental setup consisted of a 25 L working volume membrane reactor equipped with a submerged hollow-fiber membrane module (PVDF, inner/outer diameter 0.5/0.4 mm, pore size = 0.1 μm, filtration area = 1.5 m^2^) (Figure 1). The reactor was equipped with an air pump (flow = 47 L min^−1^, installed electric power 40 W) and a ceramic diffuser below the membrane module. The latter was connected to a diaphragm pump (flow = 2.5 L min^−1^, installed electric power 20 W). The system was equipped with pressure and temperature indicators. 

### 2.4. Effect of Additives on Membrane Reactor Performance

The membrane reactor was operated with and without the use of additives to evaluate the effect on membrane hydraulic performance and permeate quality. The additives consisted of either powder activated carbon (Sigma Aldrich, St. Louis, MO, USA), powder zeolite (Olympus-minerals, Thessaloniki, Greece), powder iron hydroxide (GEH Wasserchemie, Osnabrück, Germany) or activated sludge (municipal wastewater plant). Each additive was tested in a different experiment (named PAC, ZEO, GEH and MBR respectively) at a constant dosage equal to 0.9 g L^−1^ inside the membrane tank. A control experiment (no use of additives) was also performed in a direct membrane filtration (DMF) mode of operation. Reactor feeding was performed in fed-batch mode using 5 L of synthetic greywater per feeding, for a total of 6 times per day, simulating water use in the wash basin or the kitchen sink. During all experiments, membrane hydraulic performance and permeate quality were evaluated. The membrane used for the study was cleaned between different experiments using tap water supplemented with 100 ppm NaOCl, until the initial permeate flux was recovered (50–54 L m^−2^ h^−1^). Transmembrane pressure was continuously recorded using a pressure gauge and it remained between 0.62 and 0.84 bar. The operational temperature varied between 15.0 and 21.5 °C.

### 2.5. Medium-Term Operation of the Membrane Reactor Using Activated Sludge

The MBR system was inoculated with activated sludge obtained from an extended aeration facility treating municipal wastewater. The activated sludge was used without prior acclimation at initial TSS concentration 0.9 g L^−1^ inside the membrane reactor, and the system was set in operation for a period of 14 days using synthetic greywater. Reactor feeding was performed in fed-batch mode using 5 L of synthetic greywater per feed, for a total of 6 times per day. The corresponding daily volume of greywater treated was 30 L giving a hydraulic retention time of 20 h and a solids retention time of 14 days (no sludge withdrawal was performed during the study period). The permeate pump was set in operation immediately upon greywater feeding and the permeate flowrate was recorded using a graduated cylinder (1 L) and a timer, until the 5 L were recovered. Influent and permeate samples were collected every second day and analyzed for pH, COD and PO_4_-P concentrations according to the Standard Methods for the Examination of Water and Wastewater [21]. The operational temperature remained on average 18.3 ± 1.0 °C.

### 2.6. Calculations

Normalized flux (*J*) was calculated according to the following equations:

(1) $$J=J_{T}\times 1.012^{\left(20-T\right)},$$

(2) $$J_{T}=Q_{P}÷A_{m},$$

where

*T* = operational temperature (°C)
*J_T_* = permeate flux at operational temperature Τ (L m^−2^ h^−1^)
*Q_P_* = permeate flowrate at operational temperature Τ (L h^−1^)
*A_m_* = membrane filtration area (m^2^)

## 3. Results

### 3.1. Frequency and Duration of Water Use at Student Residences

The data collected by the participating students revealed high variability in the duration of water usage, ranging from 20 to 210 s per use of the wash basin, 35 to 240 s per use of the kitchen sink and 280 to 613 s per use of the shower. Considering also the frequency of water usage (see Table 1), it becomes evident that greywater inside the household is generated within 15 to 45 min. This is considered of major importance for the design of micro-scale greywater treatment systems. 

The quantity of greywater generated from the wash basin and the kitchen sink, was between 2–21 and 4–24 L per use, and on average 28 and 25 L d^−1^ (per inhabitant) respectively. Indeed, the quantity of greywater generated from the wash basin or the kitchen sink, was comparable to that of freshwater used for toilet flushing (on average 22 L d^−1^). Finally, consumption of surfactant products in the wash basin (cream soap) and the kitchen sink (liquid dishwasher detergent) was equal to 2.5 ± 0.5 and 3.7 ± 0.9 mL per use, corresponding to 0.28 ± 0.07 and 0.31 ± 0.13 mL L^−1^ greywater, respectively.

### 3.2. Effect of Additives on Membrane Reactor Performance 

**Figure 2:**
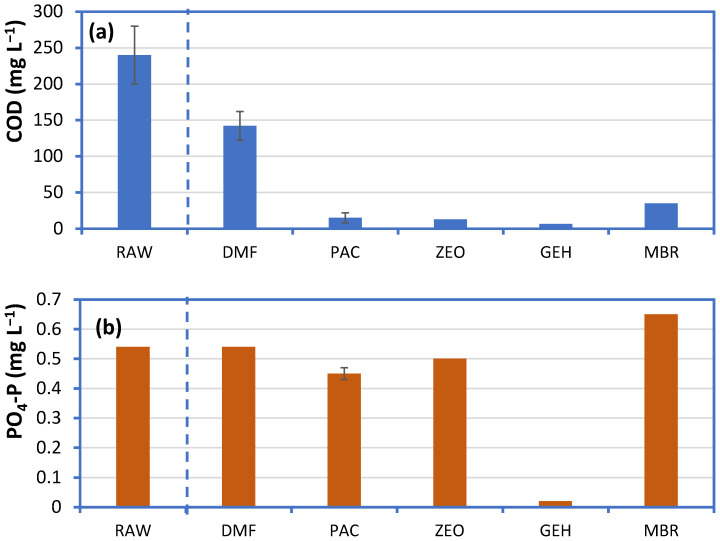
Quality of the permeate (**a**) COD and (**b**) PO_4_-P during short-term operation of a membrane reactor with different additives, treating 60 L synthetic greywater (RAW) (DMF: Direct membrane filtration; PAC: Powder activated carbon; ZEO: Powder zeolite; GEH: Powder iron hydroxide; MBR: Activated sludge). Additive concentration was equal to 0.9 g L^−1^ in all cases.

The use of additives improved permeate quality in the short-term compared to the direct membrane filtration mode of operation (Figure 2). The use of GEH was efficient both for COD and phosphorus removal from greywater, and the respective concentrations in the permeate were lower than 20 and 0.05 mg L^−1^ respectively. High permeate quality in terms of COD was also recorded for the PAC, ZEO and the MBR system, but in these cases, phosphorus remained almost unaffected. In comparison, direct membrane filtration of greywater (DMF) resulted in high permeate COD between 120–170 mg L^−1^. Similarly, a dramatic increase of permeate COD (from 15 to 170 mg L^−1^) was recorded by the fourth day of PAC system operation (data not shown), indicating that the material became saturated.

**Figure 3:**
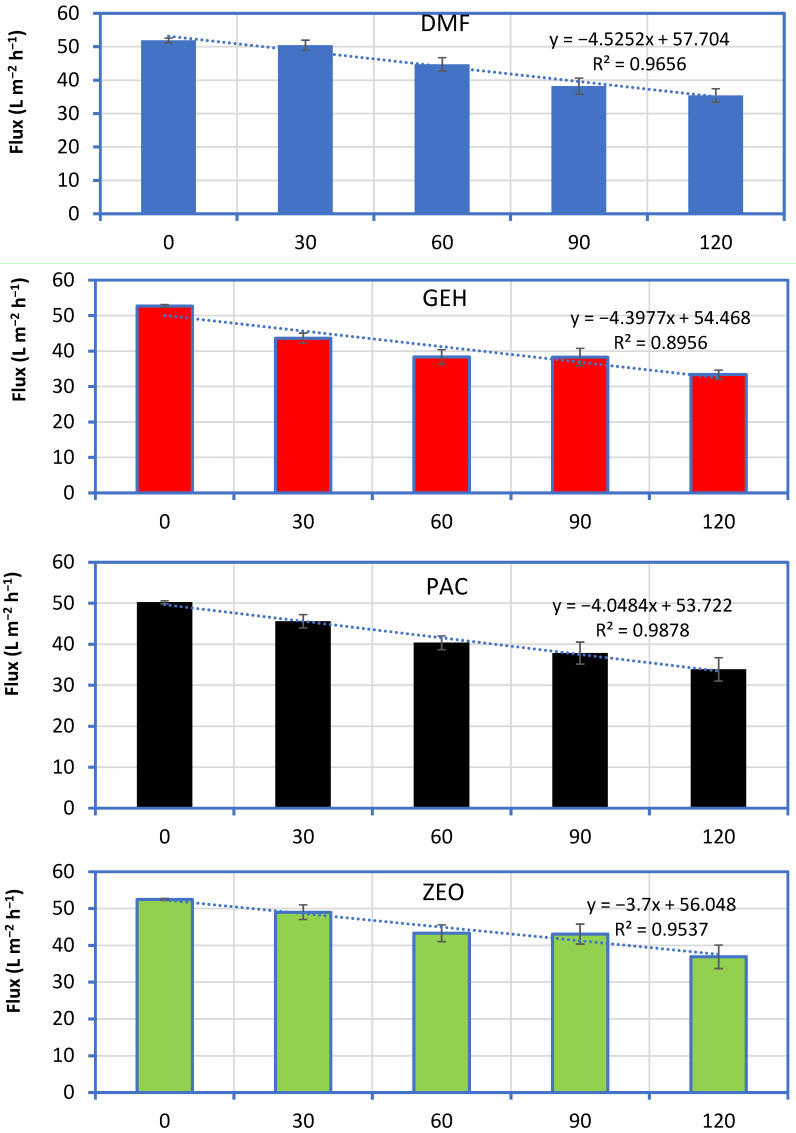

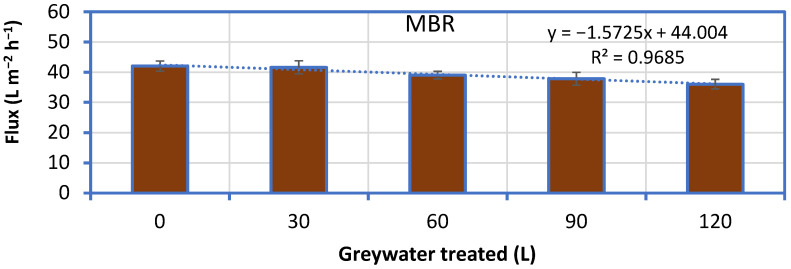
Normalized permeate flux as a function of greywater volume treated during short-term operation of a membrane reactor with different additives, treating synthetic greywater (DMF: Direct membrane filtration; PAC: Powder activated carbon; ZEO: Powder zeolite; GEH: Powder iron hydroxide; MBR: Activated sludge).

Based on the results presented in Figure 3, the permeate flux of all systems showed a linear decrease during continuous operation with greywater. The rate of flux decline was on average 4.5, 4.4, 4.0, 3.7 and 1.6 L m^−2^ h^−1^ per 30 L of greywater treated, for DMF, GEH, PAC, ZEO and MBR systems, respectively. Indeed, no significant improvement of membrane hydraulic performance was recorded when PAC, GEH or ZEO were added inside the membrane tank, despite that these additives showed negligible membrane fouling when operated with tap water alone. On the contrary, the use of activated sludge decreased clean water flux from initially 51.2 (±0.3) to 48.7 (±1.0) and 42.0 (±1.7) L m^−2^ h^−1^, with increasing activated sludge concentration, from 0 to 0.45 and 0.90 g L^−1^, respectively, which was not the case when greywater was used as a feed.

### 3.3. Medium-Term Operation of a Micro-Scale MBR System

**Figure 4:**
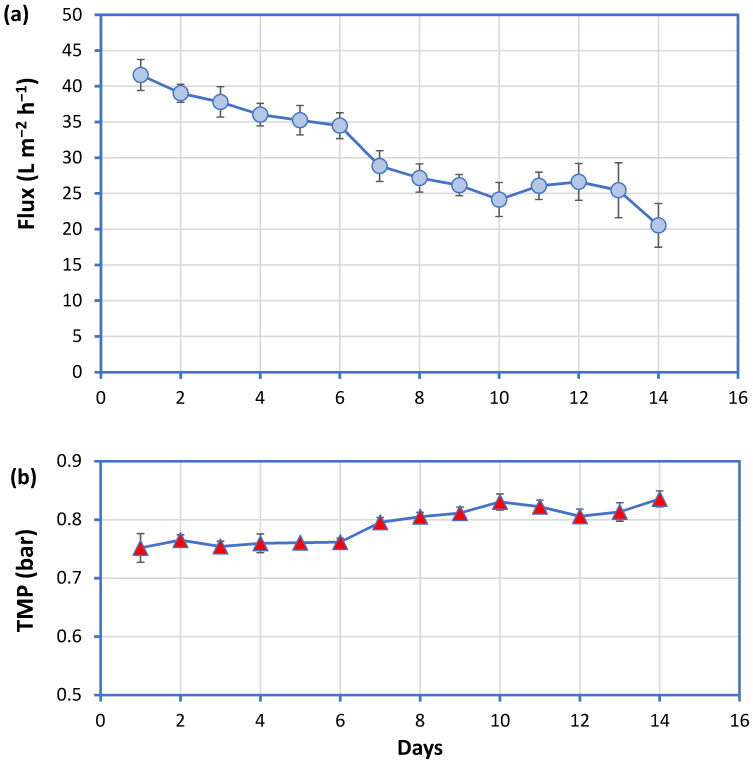
(**a**) Normalized permeate flux and (**b**) transmembrane pressure (TMP) during continuous operation of a micro-scale MBR system treating synthetic greywater.

The evolution of the permeate flux and the transmembrane pressure (TMP) of the MBR system during medium-term operation are given in Figure 4. Permeate flux decreased from initially 42 L m^−2^ h^−1^ and remained relatively constant at 25 L m^−2^ h^−1^. The TMP was equal to 0.76 bar during the initial 6 days of operation, and then increased to 0.80–0.85 bar. The MBR permeate was characterized by low COD concentration (on average 28 ± 14 mg L^−1^) which remained constant during the study period. Phosphorus concentration on the contrary displayed a gradual decrease from 1.25 to 0.35 and 0.05 mg L^−1^ during the third, fifth and seventh days, respectively, and then remained at low levels (0.04–0.05 mg L^−1^). The suspended biomass concentration inside the bioreactor was 0.4–0.6 g L^−1^, despite that the system was inoculated with 0.9 g L^−1^, indicating that a significant fraction was entrapped onto and within the hollow-fiber membrane module. This was also evidenced by macroscopic observations.

## 4. Discussion

### 4.1. Design Considerations for Household Greywater Treatment

Greywater flowrate and variability is vital for the design of micro-scale treatment systems [16]. While previous studies reported that the majority of greywater flows occur between 7–10 am and 5–10 pm, data about the exact duration of water use and the quantities of greywater generated per use for different household activities are scarce. The results of this work demonstrate that greywater at student residences was generated within 15 to 45 min per day and the average quantity of water consumed at the wash basin and the kitchen sink was 9 ± 5 and 13 ± 6 L per use. However, more research is required on this topic, since flow rate variations in family houses may vary significantly compared to the examined student residences. The total quantity of greywater generated by the participating students was within the same range reported in the literature. Shaikh and Ahammed [16] calculated greywater production on average 71 L d^−1^ per inhabitant for high income countries, which consisted between 60–75% of the total household water consumption [14,22,23]. 

### 4.2. The Role of Additives for Direct Membrane Filtration of Greywater 

Direct membrane filtration can significantly decrease the greywater organic content; however, an important fraction (30% to 50% of COD) still remains in the permeate. The greywater colloidal particles are retained inside the membrane tank and contribute to cake layer formation, while soluble organics will gradually narrow and block the membrane pores [24,25]. To this end, additives can alleviate membrane fouling (at least at short-term) due to the adsorption of soluble compounds [26]. Based on the results of our study, the permeate flux was not significantly improved when PAC, ZEO or GEH were added inside the membrane tank, although COD removal was higher than 90%. Besides, adsorbents are characterized by a limited adsorption capacity, which can adversely affect long-term system performance. The adsorption capacity of zeolite and activated carbon was recently reported as 26 and 32 mg COD g^−1^, respectively [12]. Zeolites entail a lower cost (200–300 € tn^−1^) compared to activated carbon (1000–2000 € tn^−1^); however, regeneration (or withdrawal and replacement) is necessary to ensure long-term efficiency. This issue can be a limiting factor for household applications where residents involvement must be minimal. Finally, regular replacement of saturated adsorbents will eventually increase the operational costs of the proposed facility.

Activated sludge can effectively decrease soluble COD concentrations, mainly due to biodegradation. This is beneficial to maintain high permeate flux and effluent quality. According to Hernandez-Leal et al. [27], who tested different greywater treatment systems, optimum performance in terms of COD and micropollutants removal was achieved by aerobic biological processes. Similarly, Ghunmi et al. [22] concluded that the most feasible greywater treatment process in terms of performance and costs, was particle separation combined with organic pollutants degradation. The membrane bioreactor in our study achieved high COD removal efficiency (85%), which was combined with a permeate flux between 40 and 25 L m^−2^ h^−1^. Similar permeate quality and permeate flux decline patterns were reported in previous MBR systems treating greywater [2,10]. The use of carriers for activated sludge immobilization and retention can minimize sludge washout, as demonstrated in the study of Jabornig and Favero [10]. Under these conditions, excess (suspended) sludge can be withdrawn from the reactor along with greywater overflow to the sewer. Considering the above, it becomes evident that the MBR system is the most promising micro-scale treatment system for further investigation and long-term studies under field conditions are necessary to examine both process efficiency, maintenance requirements and evaluate sound and odor nuisances.

### 4.3. Economic Feasibility and Comparison with Previous Studies

A comparison of different household greywater treatment systems is given in Table 2. Based on current market prices, the experimental setup used in our study entail a capital cost (CAPEX) of around 300 €. Operational expenses (OPEX) include the annual replacement of the membrane module (estimated cost at 60 € per year) and the electricity consumption which is considered low (<200 kWh or 20 € per year). Juan et al. [28] designed a compact greywater treatment system for possible installation inside the bathroom. It comprises of a greywater collection tank with pump, a series of polypropylene and activated carbon filters and a (purified) water storage tank. The recovered water can be used for toilet flushing and gardening-irrigation purposes and the authors claimed that it was possible to decrease freshwater consumption by up to 50%. A multi-stage treatment system was proposed by Kant et al. [11] consisting of several filtration cartridges (coarse filter, microfilter, activated carbon, ultrafiltration, reverse osmosis and ultraviolet) for in-house water reuse. The capital cost of the proposed facility system was 800 €; however, no data were given for maintenance and cartridge replacement expenses. Jabornig and Favero [10] provide data for an on-site micro-system for greywater treatment based on the membrane bioreactor technology. The facility was designed for a four-member family treating around 200 L greywater per day. It was set in operation for a period of 10 months using synthetic greywater. The beneficial effects of using the MBR technology included high removal efficiency of pathogens and organics. However, the overall volume occupied by the MBR was 200 L, enabling installation inside the apartment rather difficult. Gross et al. [29] developed a constructed wetland for household greywater treatment. The system was operated using highly polluted greywater (COD = 850 mg L^−1^) and a significant fraction of COD remained at the effluent (157 mg L^−1^). Finally, Jefferson et al. [30] highlight the use of commercially available greywater treatment systems in the UK consisting of microfilters and disinfection. 

Based on the data provided on Table 2, it is evident that the costs for purchase and installation of household greywater treatment systems vary from 300 to 1100 €. The operational expenses were between 70 and 165 € per year and included mainly the replacement of filter cartridges followed by electricity consumption. The latter in most of the cases was lower than 3 € per month. Considering the above, the cost of water recovered by the proposed technologies vary between 2 and 5 € m^−3^ (between 25% and 50% due to CAPEX). Clearly, the purchase and installation of such greywater treatment systems can be incentivized in order to leverage their penetration at household level. In comparison, an MBR system for 25 PE entail a CAPEX of around 0.85 € m^−3^ and an OPEX of 0.40 € m^−3^ [6]. Despite the low cost of freshwater in some regions, the benefits from broad greywater reuse becomes evident at municipality level and in regions suffering from water scarcity [2,3,4,5]. 

## 5. Conclusions

A micro-scale membrane reactor was designed and operated using synthetic greywater. The use of additives (PAC, ZEO and GEH) improved permeate quality compared to the direct membrane filtration mode of operation; however, no significant improvement of membrane hydraulic performance was recorded. The use of activated sludge (MBR) resulted in high COD and phosphorus removal efficiency, while permeate flux remained relatively constant. Compared to micro-scale systems reported in the literature, the proposed MBR technology entails low capital and operational costs rendering the process feasible at household level.

## Figures and Tables

**Table 1:** Duration, frequency and quantity of water use at student residences.

	Min	Max	Average	Stdev
**Duration of water use (s) per use**				
Wash basin	20	210	92	48
Kitchen sink	35	240	133	56
Shower	280	613	438	108
**Frequency of water use per day**				
Wash basin	2	4	3	1
Kitchen sink	1	3	2	1
Toilet	2	5	4	1
Shower	1	2	1	0
**Quantity of water use (L) per use**				
Wash basin	2	21	9	5
Kitchen sink	4	24	13	6
Toilet	6	6	6	0
Shower	28	61	44	11
**Quantity of water use (L) per day**				
Wash basin	18	43	28	8
Kitchen sink	13	42	25	9
Toilet	12	30	22	5
Shower	28	61	44	11

**Table 2:** Technical and economic comparison of household greywater treatment systems.

Technology	Foot-Print	CAPEX (€)	OPEX (€/year)		Source
Micro-scale MBR	25 L	300	60 20	Membrane replacement Electricity consumption	This study
Cartridge filtration (polypropylene, activated carbon)	Not reported	450	130 35	Filter replacement Electricity consumption	[28]
Vertical flow constructed wetland	1000 L	500	80	Maintenance and labor	[29]
Cartridge filtration (microfiltration, ultrafiltration, reverse osmosis, UV)	250 L	880	Not calculated		[11]
Coarse filtration and disinfection	Not reported	550–1100	Not reported		[30]
MBR	Not reported (2 m^3^/d or 25 PE)	6000	120 100 80	Disinfectant Electricity consumption Labor (per hour)	[6]
